# Lived experiences of couples in a relationship where one partner is diagnosed with a mental illness

**DOI:** 10.4102/curationis.v42i1.2015

**Published:** 2019-09-19

**Authors:** Andile G. Mokoena, Marie Poggenpoel, Chris Myburgh, Annie Temane

**Affiliations:** 1 Department of Nursing Science, University of Johannesburg, Johannesburg, South Africa; 2 Department of Educational Psychology, University of Johannesburg, Johannesburg, South Africa

**Keywords:** couples, lived experiences, mental illness, partners, relationship

## Abstract

**Background:**

A partner with mental illness can be challenging in a couple’s relationship. Mental illness brings about disintegration in the relationship because the partner without mental illness takes on more responsibilities than before. The partner without mental illness can be subjected to multiple risks, including stress and burden of care. The lived experiences of couples in a relationship where one partner is diagnosed with a mental illness is an under-researched area of mental health.

**Objectives:**

To explore and describe the lived experiences of couples in a relationship where one partner is diagnosed with a mental illness.

**Method:**

A qualitative, descriptive, exploratory and contextual research design was utilised. A purposive sampling method was used to sample participants for this study. Five couples, where one partner was diagnosed with a mental illness, participated in the study. Ten in-depth, individual, phenomenological interviews were conducted to provide rich descriptions of the couples’ experiences. Data were analysed using thematic analysis. An independent coder assisted with the data analysis. A consensus discussion was held between the independent coder and the interviewing researcher to agree on the identified themes.

**Results:**

Four themes with categories emerged from the data analysis: couples experienced changed social roles in their relationship, emotional upheaval was experienced by the individual partners in the couple relationship, interpersonal distance was experienced in the couple’s relationship and a changed relationship with the self was experienced by the individual partners in the couple relationship.

**Conclusion:**

The results concluded that couples experienced that the presence of mental illness in their relationship adversely affects the relationship, thus emphasising the need to empower the couples dealing with challenges of being in a relationship where one partner is diagnosed with a mental illness.

## Introduction

A partner who has been diagnosed with a mental illness can be challenging in a couple’s relationship. Mental illness brings about disintegration in the relationship because the partner without mental illness takes on more responsibilities in the relationship, whether for him or herself, for the mentally ill partner or for any children in the relationship (Crowe 2004:309). The partner without mental illness can be subjected to multiple risks, including stress and burden of care. Rowe and Morris (2012:328) concur that a couple in a relationship where one partner is diagnosed with a mental illness are more likely to experience relationship dysfunction and dissolution. The partners are also at risk for relationship distress and caregiver burden.

Partners in a relationship depend on one another for survival and maturation of their relationship. However, such maturation can be diminished when one partner has a mental illness. Such challenges have been shown to result in disintegration of the couple’s relationship (Bowen 1994:174–176) because they tend to share most aspects of their lives and spend most of their time together. Couples in late-life marriages tend to rely on each other to meet the daily challenges they experience (Walker & Luszcz 2009:468). If there is a challenge in the couple’s relationship, the equilibrium is shifted as attention is also shifted. Life seems challenging when a spouse becomes burdened by illness, particularly when there is a mental illness in the relationship.

The presence of mental illness in the relationship affects the primary function of couples, namely maintaining adult personalities. The seminal work of Bowen (1994:174–176) indicates that a couple is a system, while Gurman (2011:281) defines a couple as a marital pair that is an inseparable subsystem within the family whose relationship can only be fully understood within a larger familial, historical and present-day context. Bowen (1994:176) reports that if one part of the system becomes disintegrated, the whole system becomes disintegrated. This disintegration affects the couple’s relationship and has psychological and social consequences. According to Lawn and McMahon (2014:260–261) and Crowe (2004:310), any illness or condition will cause a change in the balance within the relationship, with the partner without mental illness trying to retain the psychological and social equilibrium. This forces the partner without mental illness to be more flexible to maintain the homeostasis in the relationship (Crowe 2004:310), which may result in anxiety in the partner who does not have a mental illness.

Bowen (1994:174–176) asserts that the key generator of anxiety in families, and for couples, is the perception of either too much closeness or too great a distance in the relationship. In addition, couples may not have the capacity to think through their responses to their relationship dilemmas, but react anxiously to perceived emotional demands. The disintegration of the couple’s relationship is precipitated by increased distance in the relationship, which leads to anxious reactions from partners who perceive emotional demands. According to Power et al. (2016:76), the complexities of relationships where one partner is diagnosed with a mental illness are unpredictable or chaotic. When a person develops mental illness, their experience of the illness, recovery and treatment inevitably affects the people with whom they are most connected. Evans, Nizette and O’Brien (2017:223) state that for some this means changes in everyday routine, causing them to shift roles. Crowe (2004:311) asserts that good communication, aided by couple counselling, will assist in resolving the anxieties experienced by the couple.

Lawn and McMahon (2014:260–261) and Crowe (2004:312–314) studied the effects of specific illnesses on couple relationships and highlighted that mental illness affects both partners, resulting in continual spousal pressure and disturbed sexual functioning. In both studies, the couples mentioned altered spousal relationships once mental illness appeared. Depression has also been mentioned as a common cause of disturbance in a couple relationship because it affects both partners’ self-esteem. The partner without mental illness assumes more responsibilities in the relationship, causing them possible conflict; this may lead to further depression, causing couples to become more insecure and detached (Antoine et al. 2018:1849; Crowe 2004:312). However, these authors offer several considerations for mental healthcare professionals in order for them to be sensitive when assisting couples in coping with mental illness in the relationship. Among these, the needs of the couple and the welfare of the relationship must be considered.

There is paucity in research about the lived experiences of a couple where one is diagnosed with a mental illness. Crowe (2004:316) avows that it is surprising how little research is reported on couples, mental illness and its challenges because mental illness is a frequent occurrence. Globally, psychiatric illnesses affect a significant percentage of the population and there is a need to explore couples’ relationships where one is diagnosed with a mental illness.

## Problem statement

Couples in a relationship where one partner has a mental illness were observed to be experiencing challenges with the daily management of their relationship. The couples seemed to experience a variety of challenges around coping with the diagnosis. These challenges included changes in their relationship, diminished communication and a decline of intimacy in the relationship. There is a lack of available research on couples and how they experience being in relationships where one partner is diagnosed with a mental illness. The researchers thus recognised a dire need to explore and describe the lived experiences of couples in a relationship where one partner is diagnosed with a mental illness.

The research question was: ‘What are the lived experiences of couples in a relationship where one partner is diagnosed with a mental illness?’

## Objectives of the study

The objectives of the study were to explore and describe the lived experiences of couples in a relationship where one partner has a mental illness.

## Definition of key concepts

*Experiences* are defined by Hole and Hawker (2004:195) as knowledge and skills gained over time. In this study, experiences refer to a couple’s knowledge and skills gained over time in a relationship where one partner is diagnosed with a mental illness.

A *couple* refers to two persons who are married (either legally or customarily), engaged or otherwise romantically paired (Merriam-Webster Dictionary 2018:1). In this study, a couple refers to two persons who are married, engaged or romantically paired in a relationship where one partner has been diagnosed with a mental illness.

A *relationship* is a way in which two or more people or things are connected, the state of being connected, or an emotional or sexual association between two people (Soanes & Stevenson 2008:1214). In this study, a relationship refers to a couple’s relationship where one partner is diagnosed with a mental illness.

*Mental illness* refers to a syndrome characterised by clinically significant disturbance in an individual’s cognition, emotion regulation or behaviour that reflects a dysfunction in the psychological, biological or developmental processes underlying mental functioning (APA 2013:20). In this study, mental illness refers to clinically significant disturbances affecting the individual’s relationship patterns.

## Research design and method

### Research design

A qualitative, descriptive, exploratory and contextual design was utilised in this study (Gray, Grove & Sutherland 2017:689; Polit & Beck 2018:401). This design was chosen to enable the researcher to gather in-depth information on couples’ experiences of being in a relationship where one partner is diagnosed with a mental illness.

### Research method

In this study, a phenomenological approach providing in-depth descriptions of the lived experiences of couples in a relationship where one partner is diagnosed with a mental illness was utilised.

### Setting

The study was conducted in a mental health institution where the partner diagnosed with a mental illness was admitted. The mental health institution is located in Pretoria, South Africa. It is classified as a specialised mental health institution with 744 beds, with a monthly bed occupancy rate of 85% – 95% (637–707 occupied beds). This mental health institution was ideal for this study as it is the only tertiary specialised institution in Pretoria.

### Population and sampling

The study population consisted of couples in a relationship where one partner is diagnosed with a mental illness. The participants were purposefully selected to provide detailed descriptions of their experiences. The sample was composed of five heterosexual couples in the age range of 22–45 years. The couples had all been in the relationship for at least 3 years.

### Data collection

In-depth, individual, phenomenological interviews were conducted with both partners in a relationship where one has been diagnosed with a mental illness. Each partner was individually interviewed to allow them an opportunity to give a true reflection of how they experience the relationship. The aim was to construct realities and examine perceptions as experienced by couples. The focus was on the interpretation of their experiences (Patten & Newhart 2018:65). The central open-ended question that was posed to all participants was ‘what is it like for you to be in this relationship?’ The interviews lasted 45–60 min, and participants signed a consent form and gave permission in writing for the interviews to be audio-recorded. Field notes were also employed to enrich the collected data (LoBiondo-Wood & Haber 2018:510). Data saturation was reached with the fourth couple; the fifth couple was thus interviewed for control, to ensure that data saturation was indeed reached. Data saturation is described as the point when the information being shared with the researcher becomes repetitive (LoBiondo-Wood & Haber 2018:510).

### Data analysis

The transcribed recorded interviews and field notes were analysed using the thematic method of data analysis (Creswell & Poth 2018:123). The interviews were transcribed along with the field notes. A protocol with guidelines was given to an independent coder, who is an experienced qualitative researcher, to carry out the thematic analysis. The interviewing researcher and the independent coder met for a consensus discussion to agree on the identified themes.

### Measures to ensure trustworthiness

Trustworthiness was adhered to using the strategies described by Lincoln and Guba (cited in Denzin & Lincoln 2018:801) as credibility, transferability, dependability and confirmability. Credibility was enhanced through triangulation of the data collection methods, namely observations, field notes and interviews. Transferability was promoted by a dense description of the participants’ demographics. A rich description of the results, supported by direct quotations from participants, was also recorded. Dependability was ensured by following a clear and thoughtful research strategy (Bless, Higson-Smith & Sithole 2013:237), and confirmability was enhanced through the use of an independent coder with experience in qualitative research.

### Ethical considerations

The researcher adhered to four core ethical principles: autonomy, non-maleficence, beneficence and justice (Dhai & McQuoid-Mason 2011:169; Terry 2015:62). Autonomy means that the researcher allowed the participants to make their own decision regarding participation in the study. After obtaining ethical clearance from the academic ethics committees of two universities, both prospective participants agreed to participate voluntarily and informed consent was obtained from both partners. Informed consent from the partner diagnosed with a mental illness was obtained when he or she was stable and able to make their own decision in terms of participating in the study. To achieve this, the researcher assessed the ability of the partner diagnosed with a mental illness to understand and communicate that understanding of participating in this study (DoH 2015:16). The mental welfare of the partner diagnosed with mental illness was continuously monitored to ensure that he or she remained fit to participate in the study. Confidentiality and anonymity were maintained by not disclosing data that directly identify the participants.

The mental health institution where the study was conducted also gave permission for the study to proceed. The ethical clearance numbers are AEC32/02/2011 and 89/2011, respectively. The institution where data were collected was not disclosed in the study, thus maintaining confidentiality and anonymity.

Non-maleficence meant that the participants were not subjected to harm in this study, thus no potential harm was anticipated. The interviewing researcher offered the participants emotional support after the interviews where emotional discomfort was noted. Additional details were also provided on how the participants could seek help.

Beneficence: The researcher discussed with the participants that there were no financial benefits to them for their participation in the study. However, their participation could benefit those who are experiencing the same challenges in their relationships, and contribute to the scientific body of knowledge.

Justice: Participants were selected according to the determined criteria for this study.

## Results

### Demographic representation of the participants

Ten participants (five couples) ranging from 22 to 45 years – five men and five women – participated in the interviews. Three of the couples were black people, and two couples were white people.

The partners with mental illness were unemployed, and they had all completed matric. Partners without mental illness were employed and some have tertiary levels of education. Table 1 gives a description of the participants’ demographic profile.

**TABLE 1:** Demographic profile of participants.

Couple no.	Wife/husband	Age (years)	Educational level	Employment status	Ethnicity	Language	Mental illness/not
Couple 1	Husband (participant 2)	28	N3 Engineering	Employed	White people	Afrikaans	Does not have mental illness
	Wife (participant 1)	22	Matric	Unemployed	White people	Afrikaans	Has mental illness
Couple 2	Husband (participant 3)	35	Matric	Unemployed	Black people	IsiZulu	Has mental illness
	Wife (participant 4)	33	Matric	Employed	Black people	IsiZulu	Does not have mental illness
Couple 3	Husband (participant 5)	29	Matric	Employed	White people	English	Does not have mental illness
	Wife (participant 6)	27	Matric	Unemployed	White people	English	Has mental illness
Couple 4	Husband (participant 8)	44	Matric	Unemployed	Black people	Setswana	Has mental illness
	Wife (participant 7)	40	N6 Vocational studies	Employed	Black people	Setswana	Does not have mental illness
Couple 5	Husband (participant 10)	34	Matric	Unemployed	Black people	Setswana	Has mental illness
	Wife (participant 9)	31	Matric	Employed	Black people	Setswana	Does not have mental illness

*Source*: Adapted from Adapted from Mokoena, A.G., 2012, ‘Facilitation of the mental health of couples in a relationship where one is challenged with mental illness’, pp. 33–34, M.Cur Psychiatric Nursing. Minor dissertation, University of Johannesburg, Auckland Park

Four themes emerged from the analysis, as shown in Table 2: couples experienced changed social roles in their relationship; emotional upheaval was experienced by the individual partners in the couple relationship; interpersonal distance was experienced in the couple relationship; and a changed relationship with the self was experienced by the individual partners in the couple relationship (Mokoena 2012:36–54). These themes are presented in detail in the sections that follow.

**TABLE 2:** Themes and categories on the experiences of couples where one partner is diagnosed with a mental illness.

Themes	Categories
Experienced changed social roles in their relationship	Experienced redistribution of household roles
	Experienced changes in the role of family provider
	Experienced strain in financial decisions
Emotional upheaval was experienced by the individual partners in the couple relationship	Partner with mental illness: experienced guilt, frustration, insecurity, interpersonal distance
	Partner without mental illness: experienced frustration, guilt related to the need to escape, fear of violence, isolation and loneliness
Interpersonal distance was experienced in the couple relationship	Experienced frequent fights
	Experienced diminished communication
	Experienced declined sexual intimacy
Changed relationship with self was experienced by the individual partners in the couple relationship	Partner with mental illness: experienced compromised role actualisation, self-absorption and undeserving spousal support
	Partner without mental illness: experienced increased responsibility

*Source*: Adapted from Mokoena, A.G., 2012, ‘Facilitation of the mental health of couples in a relationship where one is challenged with mental illness’, pp. 35–38, M.Cur Psychiatric Nursing. Minor dissertation, University of Johannesburg, Auckland Park

#### Experienced changed social roles in their relationship

Couples experienced distribution of household roles, a change in the role of family provider and strained financial decisions. The participants described that they experienced social role changes when the partner without mental illness began to assume more household responsibilities. The partner who had a mental illness was unable to meet expectations regarding role fulfilment. This is supported by the quotes below:

‘I find it difficult for me to do what I know is expected from me to do … I neglect that aspects of my responsibility.’ (Participant 9, 34 years old, Husband)

‘It was difficult for me to think of my role as a man in this relationship.’ (Participant 7, 44 years old, Husband)

The participants described the impact that the mental illness had in the way they dealt with financial situations, as well as the strain they experienced with financial decisions:

‘It pushes out a lot of money, I mean medicines and doctors or anything like that are not, they are not cheap in our days.’ (Participant 4, 33 years old, Wife)

‘And then we end up fighting over it, it’s not enough money.’ (Participant 5, 29 years old, Husband)

The challenges described by the couple extended to changes in the role of the family provider who, in this case, was the partner without a mental illness.

‘I’m always working and this and that and I don’t spend enough time with her as well because of it.’ (Participant 5, 29 years old, Husband)

#### Emotional upheaval was experienced by the individual partners in the couple relationship

The participants described the impact of mental illness on their relationship as an emotion-evoking experience where they went through a range of emotions. Mental illness in the relationship negatively affected both partners. The partner with mental illness experienced guilt, frustration, insecurity, mistrust and interpersonal distance.

‘I do feel insecure. I feel insecure most of the times because I don’t get reassurance of our relationship or maybe I don’t see it.’ (Participant 9, 34 years old, Husband)

‘It hurts me so bad. It’s very bad; it makes me feel I’m the bad person in this relationship.’ (Participant 7, 44 years old, Husband)

A common view among the partners without mental illness was frustration, guilt related to the need to escape the caregiving role, fear of violence, isolation and loneliness.

‘There is no one that you can talk to about it.’ (Participant 5, 29 years old, Husband)

‘Errhh stressful, it’s hard to cope with and if you don’t have the guts to do it then you shouldn’t be in a relationship like that.’ (Participant 6, 27 years old, Wife)

‘Frustration like it will make me snap at him a lot and with me snapping …’ (Participant 4, 33 years old, Wife)

#### Interpersonal distance was experienced in the couple relationship

The partners described their experiences in the relationship as being different from what it was before discovering that one partner had a mental illness. The partners described constant fighting, invoked by triggers such as the nature and demands of the mental illness, and perceived lack of partner understanding and engagement. The affected partners mentioned that, at times, their partners do not give them attention.

‘I do, it feels a lot of times like I’m neglecting her.’ (Participant 5, 29 years old, Husband)

‘We drift further and further apart and nothing helps to bring us back to laughing mode anymore.’ (Participant 7, 44 years old, Husband)

‘Sometimes we don’t understand each other.’ (Participant 5, 29 years old, Husband)

The mentally ill partner displayed decreased communication and sexual intimacy, which was exacerbated by constant fights within the relationship.

‘There is lack of communication between us, we don’t understand each other.’ (Participant 3, 35 years old, Husband)

‘We are not sexually active any longer and we just, it has changed a lot.’ (Participant 1, 28 years old, Husband)

#### Changed relationship with the self was experienced by the individual partners in the couple relationship

The partner who had a mental illness experienced a sense of failure related to compromised role actualisation. The participants with a mental illness described this as being self-absorbed because of the attention that is now directed to them in the relationship.

‘I feel less of a man because she is providing for me. She is doing everything of me and I can only imagine how it feels for her.’ (Participant 7, 44 years old, Husband)

A partner not diagnosed with mental illness experienced an increased sense of responsibility. This was as a result of having to carry out almost all the household tasks.

‘It’s quite a bit because she sits around all day while I work. You know I’ve got two jobs … basically just to pay all the bills and just keep us alive at the end of the day.’ (Participant 5, 29 years old, Husband).

## Discussion

Mental illness had a negative impact on the relationship between couples who participated in this study. According to Checton et al. (2015:258), mental illness interferes in couples’ lives if the ill partner becomes less active in the household and with social responsibilities, or if the caregiving partner experiences a burden, such as physical exhaustion and mental stress. Having a partner with mental illness means an unpredictable or chaotic life for the family. For some, this means daily routine changes, causing them to shift roles (Power et al.2016:76).

In a study conducted by Lawn and McMahon (2014:260–261), participants described the continual emotional pressure they experience each day, together with the absence of emotional support. This was found to be similar to the findings of the present study. Ng et al. (2017:13) also confirmed that taking care of a person with mental illness is a stressful experience that may be harmful to one’s own mental health. Partners in such relationships suffer emotional discomfort as they must tolerate the most arduous caregiving tasks individually.

Partners diagnosed with a mental illness often experience significant distress related to mental illness. They report anger, anxiety, depression and guilt, which is similarly associated with chronic caregiver stress (Antoine et al. 2018:1843). A study conducted by Trondsen (2012:179) revealed that participants expressed strong feelings of fear as a fundamental aspect of their lives, mainly as a consequence of the rapid emotional shifts and exacerbations of mental illness. Living with a partner with mental illness is associated with psychological distress and marital strain (Rosand et al. 2012:66). This view is also supported by Falconier et al. (2014:221–235), who confirm that stress has various effects on relationship satisfaction.

Couples identify patterns in behaviours such as passive aggression, avoiding interpersonal conflict and waiting to be loved (Antoine et al. 2018:1843). In a study conducted by Lawn and McMahon (2014:260–261), couples mentioned an altered spousal relationship coinciding with the onset of mental illness. Despite their commitment to the relationship, couples described high levels of isolation and feelings of extreme loneliness, verging on dissociation and anomie that engulfed them.

Chronic illness has an impact on a relationship in terms of its meaning and function, including sexual functioning. There is, therefore, a perceived inability for the partner with mental illness to meet the expectations of their partners in the relationship, including sexual functioning. Symptoms of mental illness prevent them from enjoying pleasurable activities. The couples thus become more detached and insecure (Antoine et al. 2018:1849).

The findings of the present study are further supported by Checton et al. (2015:264), who confirm that the presence of mental illness interferes with the partners’ perceptions of receiving support from the partner who is diagnosed with a mental illness. This hampers their ability to engage in collaborative coping. Checton et al. (2015:264) describe that the experience of mental illness is associated with reduced marital satisfaction, which is associated with adjustment and health.

The present study revealed that partners with mental illness feel isolated and misunderstood by their partners. Such relationships are experienced as a trigger to interpersonal distance. Similarly, in another study, it was found that partners with mental illness reported being unable to meet their established obligations and they experienced social exclusion (Van der Saden et al. 2014:710–717).

## Limitations of the study

Challenges were experienced with partners who did not have a mental illness, as some were uncomfortable to continue sharing their personal experiences. This had no direct impact on the study as the interviewing researcher recruited other participants who were willing to participate.

## The implication for clinical practice

Couples should be empowered to deal with the emotional distress that comes with the responsibilities of being in a relationship where one partner is diagnosed with a mental illness. Psycho-education and problem-solving skills can be of use in helping couples with such changes, as well as a division of labour within the household. The partner without the mental illness should receive emotional and social support (Checton et al. 2015:258; Van der Saden et al. 2014:716) as it restores a state of homeostasis in the relationship.

The partner who is diagnosed with a mental illness may need ongoing spiritual and psychological support, which should be complemented by emotional support. Couple counselling should also be implemented to address concerns for both partners to resolve marital difficulties, as well as psycho-education to provide the partner with mental illness general information about his or her illness. The partner should also receive information on available resources for stress management. Spousal involvement should be encouraged. Couples’ therapy may teach the couple how to respond better to unmet needs when these occur in the relationship. Psychiatric nurses need to be encouraged to engage their own self-reflection and consider their own biases about the couples to provide effective support (Sauerheber, Nims & Carter 2014:231–239).

Relationship education should be provided to assist the couples in coping with a partner who is diagnosed with a mental illness. This will assist the couple to gain knowledge and skills to increase the chances of them engaging in a healthy and stable relationship (Sprenkle 2012:15–16). Couples should be engaged in sex therapy and marital therapy in severe cases of relationship or sexual difficulties. Discussions should take place that address the challenges of sexual infrequency in the marital relationship. Therapy and sex education may lead to increased relationship satisfaction and intimacy (Kennedy & Gordon 2017:319).

Ongoing spousal support should be encouraged. Empathy demonstrated by the psychiatric nurse will be useful in helping the couple to feel understood and less alone. Empathy and empathic communication are essential elements of successful relationships. Therefore, a focus on empathy in couple counselling could increase marital satisfaction (Schmidt & Gelhert 2017:27). Further research is required to determine the ways in which the mental health of couples in a relationship where one partner is diagnosed with a mental illness can be improved.

## Conclusion

The findings revealed that the couples experienced that the presence of mental illness in their relationship adversely affects the relationship because most things change in the relationship. The partner without mental illness bears the burden of having to care for his or her partner who is diagnosed with a mental illness. It also impacts the partner who is diagnosed with mental illness because he or she feels demeaned having to be provided for and taken care of. Thus, the couples need to be empowered to deal with the changes that come with being in a relationship where one partner is diagnosed with a mental illness.
